# Ultrasonographic Evaluation of The Effects of Orthodontic or Functional Orthopaedic Treatment on Masseter Muscles: A Systematic Review and Meta-Analysis

**DOI:** 10.3390/medicina55060256

**Published:** 2019-06-07

**Authors:** Romeo Patini, Patrizia Gallenzi, Roberta Lione, Paola Cozza, Massimo Cordaro

**Affiliations:** 1 Fondazione Policlinico Universitario A. Gemelli IRCCS, Roma, Università Cattolica del Sacro Cuore, Institute of Dentistry and Maxillofacial Surgery, 00198 Rome, Italy; patrizia.gallenzi@unicatt.it (P.G.); massimo.cordaro@unicatt.it (M.C.); 2 Department of Clinical Sciences and Translational Medicine, University of Rome “Tor Vergata”, 00133 Rome, Italy; robertalione@yahoo.it (R.L.); paolacozza@alice.it (P.C.)

**Keywords:** orthodontics, masticatory muscles, ultrasonography, myofunctional therapy, diagnosis

## Abstract

*Objectives*: This review investigated the effects of orthodontic or functional orthopedic therapy on masseter muscle thickness through the use of ultrasonography (US) in growing subjects when compared with untreated subjects. *Materials and Methods*: This review systematically assessed studies that investigated growing subjects undergoing orthopedic therapy for the correction of malocclusion of vertical, sagittal and transversal plane. Electronic databases (CENTRAL, MEDLINE-PubMed, Scopus and Web of Science) were searched up to February 2019, including available RCTs and CCTs, without language restrictions. The primary outcome was the effect of orthopedic or functional treatment on masseter muscle thickness. The risk of bias of included studies was assessed through the Newcastle-Ottawa quality assessment scale with the aim of defining their methodological quality. A random-effects meta-analysis analyzing mean differences with 95% confidence intervals was used for quantitative analysis. *Results*: The search retrieved 749 titles, but the studies selection resulted in a final sample of 5 CCTs. The studies retrieved data from 233 children (age range: 5–22 years) and were conducted at university dental clinics. Children were treated for Class II malocclusion, increased vertical dimension or lateral cross-bite variably with rapid or slow maxillary expansion, twin block, bite block, mandibular activators, quad helix, alone or in combination. Risk of bias was assessed as medium for three studies, low for one and high for another. The meta-analysis determined that at the end of orthopedic or functional treatment masseter muscle thickness, measured through the use of US, is significantly reduced (MD −0.79 mm; 95% CI −1.28 to −0.31). The reduction in muscle thickness, therefore, could be considered an indicator for the evaluation of the success of therapy with orthodontic appliances. *Conclusions*: Although the meta-analysis revealed that US could be considered a less invasive and effective method to evaluate the masseter muscle thickness, single-blinded RCTs, are required to confirm US reliability in this field of application. This review was registered on PROSPERO with the following registration number: CRD42018068402.

## 1. Introduction

Maxillofacial morphology is significantly affected by masticatory muscle function. Thicker masseter muscles, in fact, have been associated with brachiocephalic subjects which are characterized by an increased length of mandibular body, a reduction of anterior facial height and a horizontal pattern of growth; facial form of individuals with less thick muscles, on the contrary, showed an association with mesocephalic patients who have a higher rate of variation in facial morphology since the muscles do not influence craniofacial growth pattern in such a determinant way [1,2]. Moreover, it has been [3,4,5] reported that the presence of an agent that undermines the balance of the neuromuscular system might lead to severe malocclusions. Given the fact that the mechanism of action of orthodontic and orthopedic appliances is basically to give movement to the jawbones, it is reasonable to think that such movement can stretch the connected muscles and that the muscles can transmit forces to skeletal and dental tissues [6]. A muscle that transfers forces inevitably increases its tension and this tension causes a reactive increase in thickness. Nonetheless few studies [3,5] have been carried out about the muscular changes during orthopedic or functional treatment in growing subjects.

In recent decades, electromyography (EMG) was considered the principal technique to evaluate the body’s homeostatic abilities to adapt to the alterations of the stomatognathic system, but the results were at times contradictory. Moreover, electromyography has been used to investigate muscular changes related to thickness even though it has been demonstrated that EMG is subject to many shortcomings and may not represent true muscular activity [7].

However, this allowed clinicians to have numerical data that consequently improved the accuracy of the diagnosis because they can be statistically evaluated, also improving therapy and follow-up phase [7].

In order to avoid the inappropriateness of EMG in the evaluation of masticatory muscle thickness, more recently some authors used the ultrasonography (US) for the analysis of the masseter muscular changes in patients with malocclusion or during the treatment of malocclusions [8,9,10,11]. The advantages of the use of US are many: first of all it is a x-ray free method that avoids ethical problems for patients (especially in pediatric age); the superficial position of the masseter muscle allows it to be used in a very simple and quick way and it has a low cost for the patient [11].

To the best of our knowledge there are no systematic reviews that analyze this aspect.

Therefore, the aim of the present review was to investigate the effects of orthodontic or functional orthopedic therapy on the masseter muscle thickness through the use of US in growing subjects when compared with untreated subjects.

## 2. Materials and Methods

The present review is reported in accordance with the guidelines of PRISMA (Preferred Reporting Items for Systematic Review and Meta-Analyses) [12].

The focused question, in the PICO format (Patient, Intervention, Comparison and Outcome), was as follows: “Does the orthodontic or functional orthopedic treatment of malocclusion cause a change in masseter muscle thickness among pediatric patients affected compared to unaffected ones, and can US detect it and therefore be used as an indicator of the success of the therapy?”

### 2.1. Protocol and Registration

This review was registered on PROSPERO with the following registration number: CRD42018068402.

### 2.2. Eligibility Criteria

#### 2.2.1. Population

Growing subjects with mixed or permanent dentition undergoing orthopedic maxillary expansion or functional treatment for mandibular advancement, or for the control of increased vertical dimension in which ultrasonography has been used as a method for evaluating changes of masseter muscle thickness.

#### 2.2.2. Intervention

The orthodontic or functional orthopedic therapy for the treatment of malocclusions of:

- vertical plane (i.e., dolichocephalic patients whose treatment could include bite-block or rapid/slow palatal expander cemented on permanent or deciduous molars);
- sagittal plane (i.e., patients with Class II malocclusion treated with twin block or any device for mandibular advancement)
- transversal plane (i.e., patients with lateral cross-bite in therapy with quad helix or rapid/slow maxillary expander).

#### 2.2.3. Comparison

Untreated growing children with skeletal transverse discrepancy, increased vertical dimension or Class II malocclusion.

#### 2.2.4. Outcomes

Net linear measurements of masseter muscle thickness as assessed on US reported before and after orthopedic intervention.

#### 2.2.5. Study Design

Randomized or non-randomized Controlled Clinical Trials (RCTs or CCTs) whose population was composed by children in order to evaluate if the eventual masseter muscle changes at the end of orthopedic or functional treatment could be detected by means of US. Excluded articles included In Vitro or animal studies, studies dealing with subjects affected by systemic diseases or orthognathic surgery, studies that assessed the changes of masseter muscles by means of electromyography, case reports, case series, review articles, abstracts, and discussions.

### 2.3. Information Sources and Search

MEDLINE-PubMed, Web of Science, Scopus and the Cochrane Central Register of Controlled Trials (CENTRAL) databases were systematically searched up to February 2019 without language or publication date restrictions. Date of the last search was: 10th March 2019.

The combination of MeSH terms and free text words used for MEDLINE-Pubmed database are as follows: (1) “masseter muscle” OR “masticatory muscles”, (2) “Class II malocclusion” OR “mandibular deficiency” OR “cross bite” OR “maxillary constriction” OR “transverse discrepancy” OR “open bite” OR “dolichofacial subjects”, (3) “orthodontics*” OR “orthopedic therapy” OR “rapid maxillary expansion” OR “functional treatment”, (4) “diagnostic imaging*” OR “echography*” OR “ultrasonography*”, (5) “assessment” OR “evaluation”, (6) ((1) AND (2) AND (3) AND (4) AND (5)).

The previous search strategy was used for Medline and then slightly modified to adapt it to other databases.

Google Scholar was consulted with the same search strategy for a partial gray literature search; in fact, only the first 100 search results were analyzed.

A supplementary manual search was performed consulting: European Journal of Orthodontics, American Journal of Orthodontics and Dentofacial Orthopedics, Angle Orthodontist and Progress in Orthodontics for articles published between their inception date and February 2019. References of all selected studies were also checked, and all corresponding authors of included articles were contacted by e-mail in order to recover unpublished articles or raw data and to include as many relevant studies as possible in the analysis.

### 2.4. Study Selection

The screening process was conducted independently and in duplicate. Two reviewers (RP and RL) screened titles and abstracts of the articles according to the inclusion criteria. Subsequently, the same reviewers performed the assessment of the full-text articles. Any disagreements were solved through discussion.

### 2.5. Data Collection Process

Information from articles was extracted using specially designed data extraction forms. The reviewers obtained and screened the full-text article for studies that apparently matched the inclusion criteria, or studies in which information reported in the title and abstract was not enough about inclusion.

### 2.6. Outcome

The primary outcome was the modifications of masseter muscle thickness after completion of orthodontic or functional orthopedic therapy. Summary measures were given as difference in means.

### 2.7. Risk of Bias in Individual Studies and Quality of Evidence

Data regarding ultrasonography were extracted using a structured form.

The Cochrane Collaboration’s Risk of Bias tool was used for assessing risk of bias of all included RCTs and the Newcastle-Ottawa quality assessment scale for case control studies for all CCTs, with the aim of define their methodological quality. A further evidence of the reliability of the data emerging from the analyses was provided using the Grading of Recommendations Assessment, Development and Evaluation (GRADE) tool.

### 2.8. Data Synthesis

Heterogeneity was assessed using Review Manager software [13]. A *p*-value of less than 0.1 was considered as a significant heterogeneity. The χ^2^ test and the I^2^ statistics were used to quantify heterogeneity and to certify if the eventual differences across the studies were compatible with chance alone.

The evaluation of the selected publications identified five studies [8,9,10,11,14] among which four [9,10,11,14] were fairly homogeneous for design, population demographics, diagnostics, study period and tool used for assessment of the primary outcome. The other study [8] was characterized by heterogeneity regarding the study design, study population demographics and data acquisition. Thus, the authors decided to include all the five studies in the systematic review, but quantitative analysis of data was performed only on the four [9,10,11,14] homogeneous studies.

The quantitative analysis of mean differences was performed using a random-effects meta-analysis for continuous variables. Results of all four studies included [9,10,11,14] were given as mean values and standard deviation.

## 3. Results

### 3.1. Results of the Search

When the electronic databases were consulted, they gave 749 titles as result. The elimination of duplicate results led to 428 titles of which 416 were removed analyzing their title and abstract. According to this process 12 full-text articles were selected. Only four studies [8,9,10,11] were included in the review. After contacting the corresponding author of two of the selected articles [8,10] one additional publication was recovered [14].

**Figure 1:**
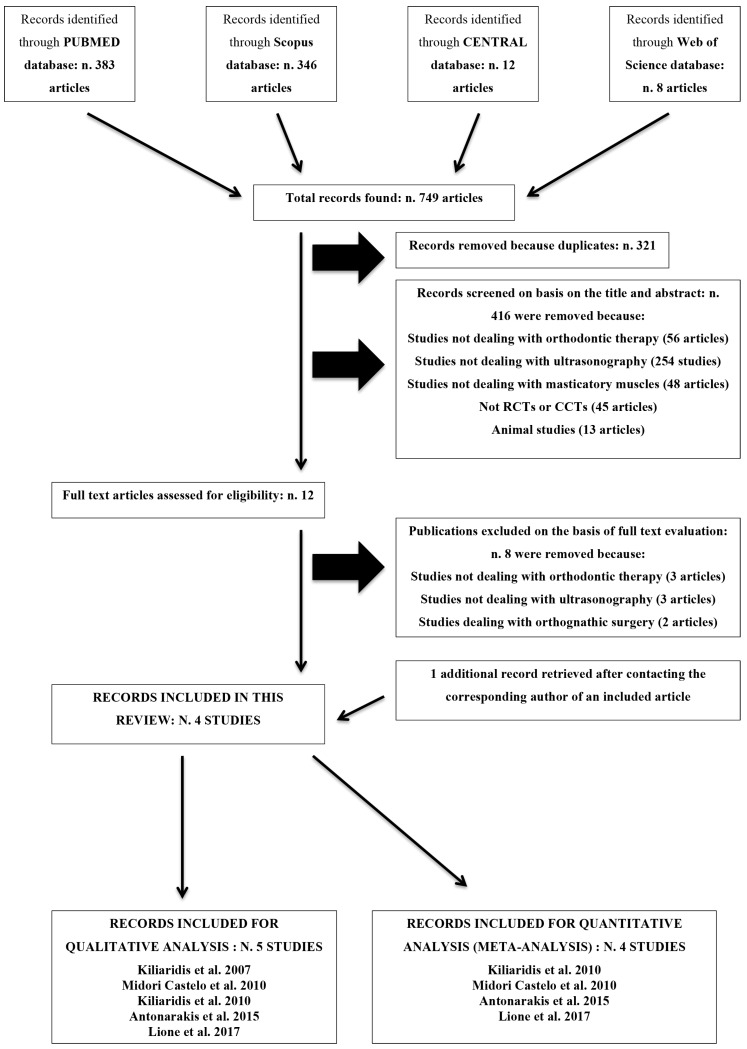
Flow chart of the screening process. RCT: randomized controlled trial; CCT: controlled clinical trial; CENTRAL: Cochrane Central Register of Controlled Trials.

In conclusion, five CCTs [8,9,10,11,14] were included in this review (Figure 1).

### 3.2. Exclusion of Studies

After full-text evaluation, three studies were excluded because the analysis was conducted on patients without need for orthodontic treatment. Three studies were excluded because they did not use US for the analysis. Two studies were excluded because they deal with orthognathic surgery. The articles excluded after the analysis of the full-text were reported in Table 1, along with the reason for exclusion.

### 3.3. Included Studies

Two studies [10,11] were carried out in Switzerland, one in Italy [14], one in Brazil [9] and one in Switzerland, Greece and Sweden according to a multicenter design [8]. Three trials had a parallel group study design [10,11,14]. In one case [9] the study had a split-mouth design and in another case [8] a third group, named “untreated group”, was provided.

All trials were conducted at University dental clinics [9,10,11,14] except for patients belonging to the untreated group of the trial with the multicenter design that were enrolled at a summer camp in Greece [8].

Five CCTs [8,9,10,11,14] were included in this review, but meta-analysis was possible only on the four homogeneous trials [9,10,11,14].

The included articles [8,9,10,11,14] analyzed the eventual use of US in evaluating masseter muscle thickness changes after orthopedic or functional therapy.

Characteristics of the five included studies [8,9,10,11,14] are reported in Table 2.

### 3.4. Characteristics of Participants

Four studies [9,10,11,14] included only pediatric patients (age range: 6–13 years); one trial [8] included patients belonging to a wider age range (12–22 years for cases and 15.2–18.2 years for controls). Patients affected by malocclusions, whose pre-treatment condition required the use of an orthodontic appliance, belonged to the test group. The control group was comprised of subjects chosen from among a sample of patients, matched for gender, age and height, with no immediate need for orthodontic treatment.

Children belonging to the test group were excluded if they: had deciduous or permanent teeth prematurely extracted or non-nutritive sucking habits, suffered for any temporomandibular dysfunction or disorders, for any type of syndrome that provokes palatal or craniofacial anomalies (like Cowden syndrome) [23] or clefting (like Pierre Robin sequence), for muscular disorders, previous facial trauma or orthodontic treatment, severe obstruction of upper airways, anterior crossbite and open or deep bite.

In addition to the excluding criteria previously reported, children belonging to the control group were excluded if they had class III malocclusion or transverse or longitudinal discrepancies.

### 3.5. Characteristics of Interventions

Kiliaridis et al. [10] used a twin-block appliance, presented by Mills and McCulloch [24], for correcting the class II malocclusion. Such appliance provides no engagement between maxillary incisors and no maxillary labial bow. The blocks were 7 mm thick. Patients had to wear the appliance full-time but not during eating and brushing. Dental casts, lateral cephalograms, US measurements of the masseter muscle thickness and body height and weight measurement were performed to all patients performed at the beginning (T1) and at the end (T2) of the therapy. US recordings were taken from patients belonging to the control group when the observation period started (T1) and ended (T2). The examiner and the technique for the US examination were the same for both groups. A real-time scanner (Pie Medical Scanner 480, 7.5 MHz linear array transducer; Pie Medical Imaging, Maastricht, The Netherlands) provided the US measurements. The masseter was scanned bilaterally on a level halfway between the zygomatic arch and gonial angle having patients seated upright with head naturally positioned. The scan plane registered the masseter muscle thickness when the muscle was relaxed and contracted, and it was positioned at 90° respect to the anterior border of the muscle and to the mandibular ramus. Patients provoked the muscle relaxation by maintaining slight interocclusal contacts, the contraction by clenching maximally in the intercuspal position. The examiner applied a light pressure during all registrations in order to avoid erroneous measurements given by the compression of soft tissues and muscles. Muscle thickness was registered to the nearest 0.1 mm of the mean of the repeated measurements since all registrations were repeated twice.

In the experimental group Antonarakis et al. [11] used only an activator, previously presented by Pfeiffer and Grobéty [25,26], throughout the 12-month study period. Clinicians instructed the patients to wear the appliance for at least half a day. The patients were inserted in a regular follow-up program of appointments for the eventual adaptation of the activator if needed. The subjects forming the control group received no orthodontic treatment during the observational period. Standard orthodontic diagnostic records were taken at the beginning of the therapy (T1) and after 12 months (T2) only from the experimental group. Clinicians also measured the maximal bite force in molar area and US masseter muscle thickness at the same time points. These last two measurements, along with measure of patients’ height, were the only records taken from control group; time points were the same as experimental group patients. The thickness of masseter muscle was measured using a real-time ultrasound scanner (FALCO 100, linear array transducer (6–8 MHz), PieMedical, Imaging BV, Maastricht, The Netherlands). All measurements were carried out by one examiner, calibrated by the operator who had developed the method. The masseter was scanned bilaterally at half the distance between the zygomatic arch and the gonial angle having patients seated upright without head support. The scan plane registered the masseter muscle thickness when the muscle was relaxed and contracted, and it was positioned at 90° in respect to the anterior border of the muscle and to the surface of the mandibular ramus in order to depict the reflection of the bone as a sharp white line. Patients provoked the muscle relaxation by maintaining slight interocclusal contacts, the contraction by clenching maximally in the intercuspal position. During all registrations the examiner applied a contact gel to the probe and a light pressure in order to avoid bias during measurements. Muscle thickness was considered as mean of the repeated measurements since all registrations were repeated twice.

Each patient treated by Lione et al. [14] followed a standardized protocol that included a rapid maxillary expansion using a palatal expander cemented on the upper first permanent molars, and then a lower removable bite-block (BB). Parents were instructed to activate the expander twice a day until achievement of the desired expansion. At the end of the expansion, the expander was maintained as a passive retainer for 6 months. After that period a removable lower BB substituted it for 12 months. The posterior bite-blocks thickness was 5 mm. Patients had to wear the appliance full-time but not during eating and brushing. For the treatment group, lateral cephalograms and US scans of the masseter muscles were performed at the beginning of the therapy (T1) and after one year of treatment with BB (T2). The control group was followed up with no treatment for one year except for an initial lateral cephalogram; in this group US recordings were taken at the same time points of study group. A calibrated examiner scanned bilaterally the masseter muscles in contraction using a real-time scanner with a linear array transducer for performing the US scans in a darkened room with the subjects seated upright without head support. Patients provoked the muscle relaxation by maintaining slight interocclusal contacts, the contraction by clenching maximally in the intercuspal position. The probe was positioned at 90° respect to the anterior border of the muscle and to the mandibular ramus. The operator applied a light pressure in order to avoid erroneous measurements given by soft tissues and muscle compression. Muscle thickness was considered as mean of the repeated measurements since all registrations were repeated twice.

Kiliaridis et al. [8] measured the thickness of the masseter muscle bilaterally. The same operator examined all the subjects. A real-time scanner (Pie Medical Scanner 480, Maastricht, The Netherlands), with a 7.5 MHz linear array transducer was used. The participants were seated upright, each with their head in a natural position with the probe positioned at 90° in respect to the anterior border of the muscle and the mandibular ramus. The operator applied a light pressure in order to avoid erroneous measurements given by soft tissues and muscle compression. Since the oblique scanning of the masseter would have provoked the increase of the muscle thickness, this potential source of error was avoided altering the angle of the probe until achievement of the best echo of the mandibular ramus. Masseter imaging and measurements were performed twice for each side putting the probe on a level halfway between the zygomatic arch and gonial angle, with patients clenching maximally in the intercuspal position. The examiner took two readings for each patient with a time interval of at least five minutes. Muscle thickness was registered to the nearest 0.1 mm of the mean of the two repeated measurements.

In the trial conducted by Midori Castelo et al. [9] the subjects of the experimental group were in treatment with a removable maxillary expander. Patients were informed about the necessity to wear the appliance all day, except for eating and brushing. The children had follow-up appointments every 15 days for the activation of the screw; the end of the treatment was considered when the unilateral crossbite was over-corrected without lateral deviations during opening and closing of the mouth. At the end of the treatment a removable plate (Hawley retainer) was used for three months as a retainer. The analyses were done three times: beginning of therapy (s1), resolution of crossbite and removal of the retainer (s2), three months later (s3). For ethical reasons, no attempts were made by the authors to organize a group composed by children affected by crossbite without treatment. The masseter muscle thickness was assessed bilaterally through the use of US (Just Vision, Toshiba Co., Minato-ku, Tokio, Japan; 56 mm/10 MHz), with the muscle in relaxation (RE) and maximal intercuspal (MI) condition. The measurements were performed three times, with the child seated upright and their head in a natural position. The recording site was determined by palpation, on a level halfway between the zygomatic arch and gonial angle, close to the occlusal plane. The final value was considered as the nearest 0.1 mm of the mean of the three repeated measurements.

### 3.6. Characteristics of Outcome Measures

All articles reported the masseter muscle thickness retrieved in children belonging to the test and the control group or in the test and control sides in case of a split-mouth design.

### 3.7. Risk of Bias in Included Studies and Strength of Evidence

The risk of bias is reported in Table 3.

Following the evaluation of the risk of bias for each study according to the Newcastle-Ottawa quality assessment scale, one trial [8] was assessed as at high risk, three trials were assessed as at medium risk [10,11,14] and one trial [9] was assessed as at low risk. The reliability of the data was evaluated using the GRADE approach (Table 4), the evidence regarding the masseter muscle thickness is considered low because of the high heterogeneity across studies and the wide confidence intervals in two of them [9,11]. 

### 3.8. Effects of Interventions

Kiliaridis et al. [10] revealed that US evaluation of masseter muscle thickness in subjects reported a decrease from an average of 10.7 mm in T1 to an average of 10.3 mm in T2. On the contrary, the control group showed a significant (*p* < 0.001) increase from an average of 10.9 mm in T1 to an average of 11.5 mm in T2.

Antonarakis et al. [11] showed that masseter thickness increased significantly from pre-treatment (T1) to post-treatment (T2) phase in the control group (from an average of 12.1 mm to an average of 12.6 mm) but not in the treatment group that decreased from an average of 12.9 mm to an average of 12.8 mm.

Results from Lione et al. [14] revealed that the outcome of this systematic review, during the study period, showed a decrease of 0.7 mm (± 0.3 mm) for the treatment group (from an average 9.6 mm ± 0.7 mm to an average 8.9 mm ± 0.7 mm) compared with an increase of 0.6 mm (± 0.3 mm) for the control group (from an average 10.3 mm ± 1.7 mm to an average 10.9 mm ± 1.7 mm).

The split mouth study design conducted by Kiliaridis et al. [8] found that masseter thickness showed an asymmetry in the untreated unilateral crossbite group. The crossbite side masseter was significantly (*p* = 0.025) thinner than on the normal side. On the other hand, patients belonging to the control group showed no statistically significant differences in all comparisons made.

The split mouth study design of Midori Castelo et al. [9] reported data regarding masseter muscle thickness in resting phase (RE) and in maximal clenching (MC) phase in unilateral crossbite patients without giving the mean of the two phases. The corresponding author of the trial was asked by e-mail about the lacking data and she kindly provided to the authors all the raw data of her analysis. Thanks to this, the authors have been able to analyze these data and get information to be included in the meta-analysis. Even though Midori Castelo et al. [9] reported data regarding two patient groups, two phases and three treatment periods, they found no evidence of masseter muscle thickness changes in all comparisons made.

**Figure 2:**
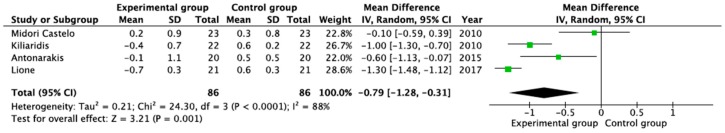
Forest plot of comparison: masseter muscle thickness changes in subjects affected by malocclusion and in healthy ones. CI: confidence intervals; SD: standard deviation.

The quantitative analysis of the four included trials (three at medium [10,11,14] and one at low risk of bias [9]) succeeded in determining that US could be an effective method for evaluating masseter muscle thickness between children unaffected by malocclusion and those affected, treated with orthodontic appliances (mean differences: −0.79 mm; 95% confidence intervals: −1.28 to −0.31), and that it could be used as a possible integrative diagnostic test (Figure 2).

## 4. Discussion

Even though orthopedic or functional therapy could lead often to satisfactory results, large variations among individuals are observed regarding skeletal and dental parameters [6,27,28]. The orthodontic treatment plan phase is not only completely based on biomechanics, but also requires a deep knowledge of the muscular environment of each patient. A deep understand of the relationship between masticatory muscles with skeletal malocclusion and craniofacial morphology may maximize the results of craniofacial orthopedics ensuring treatment stability [29,30,31]. It has been speculated that masticatory muscle functional capacity could play a role in the different response to orthopedic or functional appliance treatment in growing subjects [32,33]. In particular, there has been reported a strong correlation between patient’s morphology of face and masseter muscle thickness and that individuals with a thin masseter have a longer face [2]. Several electromyographic studies have been conducted with the aim of investigating the muscle activity during treatment with orthodontic appliances; such studies aimed at intercepting modifications of masseter thickness and at giving them a prognostic positive or negative value, but the results are sometimes contradictory [34,35,36]. These contradictions may be related to the fact that EMG is subject to many shortcomings and, as has been suggested, may not represent the real muscular activity [4].

Masseter muscle thickness has been measured by various techniques including computed tomography (CT), magnetic resonance imaging (MRI), and US scanning.

Lee et al. [21] investigated the use of CT for evaluating masseter muscle changes following orthognathic surgery; even though this method can be considered as the one giving the most well-defined images of muscular structures it should be avoided in a population of growing subjects for ethical reasons. In fact, as demonstrated by some authors [37] MRI is effective in showing soft components of head and neck region, but its application is currently limited to the study of masticatory activity and there is no evidence of its use during orthodontic treatment [38,39].

In recent years, less invasive diagnostic methods like stereophotogrammetry and US have been extensively used in the evaluation of masticatory muscle and soft tissue changes caused by orthodontic therapy for the multiple advantages over CT and MRI; in fact, many studies with these techniques have been conducted on growing subjects [8,9,10,11,14,40].

The purpose of this systematic review was to evaluate the effectiveness of US in defining the masseter changes in terms of thickness among growing subjects unaffected by malocclusion and those affected, treated with orthodontic appliances. The meta-analysis of the four included CCTs [9,10,11,14] pointed out, therefore, that US could be used as a reliable, noninvasive diagnostic exam. Moreover, it highlighted that the increase of masseter thickness seems to negatively contribute to the prognosis of the treatment with orthodontic appliances; the decrease, on the contrary, would have a beneficial effect.

The systematic review included five CCTs [8,9,10,11,14], none of which was randomized. All the selected studies [8,9,10,11,14] reported a decreased masseter muscle thickness at the end of orthopedic craniofacial intervention in children, when compared with untreated subjects who showed thicker masseter muscles at the end of the follow-up. Nevertheless, in one of the included studies [9] the thickness reduction of masseter muscle was not statistically significant but a significant reduction in thickness was reported for temporalis muscle. Such evidence could be explained considering that the study enrolled patients in deciduous dentition using a slow palatal expander for treating lateral cross-bite; probably only temporalis muscle plays a relevant role in the generation of that type of malocclusion. The reduction in the masseter size is probably due to decreased functional activity and to occlusal stability. The methodology assessment of these studies revealed that one was considered at high risk of bias [8], one was considered at low risk [9] and that the other three studies were all considered at medium risk [10,11,14]. The general lack of a strongly rigorous methodology suggests that the findings of this review should be interpreted with caution.

In fact, considering the medium level of risk of bias of three of the studies [10,11,14] included in the meta-analysis and the limited number of patients enrolled, the evidence can only mildly establish that US is able to evaluate masseter muscle changes in terms of thickness in growing subjects affected by malocclusion, treated with orthodontic appliances. For these reasons, further studies should be carried out to confirm such application of US.

## 5. Conclusions

The results of the present meta-analysis suggest that US could be used as a reliable and noninvasive diagnostic exam in the evaluation of the masseter muscle thickness. Such thickness could contribute to a negative or positive prognosis of the treatment with orthodontic appliances.

Thus, clinicians should design larger, at least single-blinded RCTs, with the aim of evaluating the efficacy of US in adjunction to EMG among both children and adults affected by malocclusion and treated with orthodontic or functional orthopedic appliances.

## Figures and Tables

**Table 1:** Table showing excluded studies after full text evaluation with rationale for exclusion.

References	Rationale for Exclusion
Becht et al. [15]	Not dealing with orthodontic therapy
Kiliaridis et al. [16]	Not dealing with orthodontic therapy
Kitai et al. [17]	Not dealing with orthodontic therapy
Antonarakis et al. [18]	Not dealing with ultrasonography
Chintakanon et al. [19]	Not dealing with ultrasonography
Dahl et al. [20]	Not dealing with ultrasonography
Lee et al. [21]	Dealing with orthognathic surgery
Trawitzki et al. [22]	Dealing with orthognathic surgery

**Table 2:** Characteristics of the included studies.

Title	Author (Year)	Type of Study	Sample Size	Mean Age at Start of Treatment in Years (SD, Range)	Mean Duration of Therapy in Months (SD, Range)	Type of Ultrasonography
Ultrasonographic thickness of the masseter muscle in growing individuals with unilateral crossbite.	Kiliaridis et al. (2007)	Controlled clinical trial	38 (17 M and 21 F) in untreated group; 18 (9 M and 9 F) in treated group; 28 (No information about sex.) in age-adjusted control group.	11.9 (NR, 8.1–17.8) in untreated group; 16.3 (NR, 12.0–22.0) in treated group; 16.1 (NR, 15.2–18.2) in age-adjusted control group.	NR	Scanner 480, 7.5 MHz. Pie Medical, Maastricht, The Netherlands.
Evaluation of changes in muscle thickness, bite force and facial asymmetry during early treatment of functional posterior crossbite.	Midori Castelo et al. (2010)	Controlled clinical trial	23 (9 M and 14 F).	5 (0.4, NR) in deciduous dentition group; 6 (0.6, NR) in mix dentition group.	13.64 (5.07, NR) in deciduous dentition group; 16.25 (5.40, NR) in mix dentition group.	Just Vision, 56 mm/10 MHz. Toshiba Co., Minato-ku, Tokio, Japan.
Masseter muscle thickness as a predictive variable in treatment outcome of the twin-block appliance and masseteric thickness changes during treatment.	Kiliaridis et al. (2010)	Controlled clinical trial	22 (8 M and 14 F) in treated group; 22 (12 M and 10 F) in control group.	9.4 (NR, 8–12) in treated group; 9.8 (NR, 8–12) in control group.	13.5 (NR, 11–17)	Scanner 480, 7.5 MHz. Pie Medical, Maastricht, The Netherlands.
Predictive value of masseter muscle thickness and bite force on Class II functional appliance treatment: a prospective controlled study.	Antonarakis et al. (2015)	Controlled clinical trial	20 in treated group and 20 in control group. *No information about sex.*	11.4 (1.3, 9–13) in treated group; 11.2 (1.9, 9–13) in control group.	12 (NR, NR)	FALCO 100, 6–8 MHz, Pie Medical, Maastricht, The Netherlands.
Evaluation of masseter muscles in relation to treatment with removable bite-blocks in dolichofacial growing subjects: a prospective controlled study.	Lione et al. (2017)	Controlled clinical trial	21 (9 M and 12 F) in treated group; 21 (9 M and 12 F) in control group.	9.9 (1.4, 8.5–11.1) in treated group; 9.6 (1.6, NR) in control group.	12 (NR, NR)	NR

M = male; F = female; NR = not reported.

**Table 3:** Review of author judgments on the sections of the Newcastle-Ottawa quality assessment scale for case control studies for each included study.

Title	Author (Year)	Selection	Comparability	Exposure	Number of Stars	Risk of Bias
Ultrasonographic thickness of the masseter muscle in growing individuals with unilateral crossbite.	Kiliaridis et al. (2007)	*	*	*	3	High
Evaluation of changes in muscle thickness, bite force and facial asymmetry during early treatment of functional posterior crossbite.	Midori Castelo et al. (2010)	****	**	**	8	Low
Masseter muscle thickness as a predictive variable in treatment outcome of the twin-block appliance and masseteric thickness changes during treatment.	Kiliaridis et al. (2010)	***	0	**	5	Medium
Predictive value of masseter muscle thickness and bite force on Class II functional appliance treatment: a prospective controlled study.	Antonarakis et al. (2015)	***	**	**	7	Medium
Evaluation of masseter muscles in relation to treatment with removable bite-blocks in dolichofacial growing subjects: a prospective controlled study.	Lione et al. (2017)	**	**	**	6	Medium

**Table 4:** GRADE Summary of Findings for Meta-Analysis on Masseter Muscle Thickness after orthodontic treatment.

Quality Assessment, Outcome: Masseter Muscle Thickness during Orthodontic Therapy						
Question: Will the Use of Orthodontic Appliances Have an Effect on Masseter Muscle Thickness?						
Number of Studies According to Meta-Analysis	Study Design	Risk of Bias	Inconsistency	Indirectness	Imprecision	Publication Bias
4 (Figure 2)	Clinical Controlled Trials	Not Serious	Serious ^a^	Not Serious	Serious ^b^	Undetected

^a^ Due to high heterogeneity across studies; ^b^ Due to wide confidence intervals.
